# Cripto-1 Promotes the Epithelial-Mesenchymal Transition in Esophageal Squamous Cell Carcinoma Cells

**DOI:** 10.1155/2015/421285

**Published:** 2015-09-17

**Authors:** Chun Huang, Wangsheng Chen, Xiaowen Wang, Jinqiu Zhao, Qian Li, Zhongxue Fu

**Affiliations:** ^1^Department of Cardiothoracic Surgery, The First Affiliated Hospital of Chongqing Medical University, Chongqing 400016, China; ^2^Department of General Surgery, The Affiliated Hospital of Luzhou Medical College, Luzhou, China; ^3^Shanghai Jiao Tong University of Medicine, Shanghai, China; ^4^Department of Respiratory Surgery, The First Affiliated Hospital of Chongqing Medical University, Chongqing 400016, China; ^5^Department of Neurology, The Fifth People's Hospital, Chongqing, China; ^6^Department of Gastrointestinal Surgery, The First Affiliated Hospital of Chongqing Medical University, Chongqing 400016, China

## Abstract

Esophageal carcinoma is a major public health problem worldwide and one of the most aggressively malignant neoplasms. Although considerable diagnostic and therapeutic progress has been made in recent years, the prognosis of EC patients still remains dismal due to high rates of recurrence/metastasis and invasion. Previous studies have demonstrated that Epithelial mesenchymal transition (EMT) is proposed as a critical mechanism for the acquisition of malignant phenotypes by epithelial cells. Several lines of evidence have shown that Cripto-1 plays an important oncogenic role during tumorigenesis by promoting EMT. The aim of our study was to evaluate the significance of Cripto-1 which plays a role in EMT and its metastasis in esophageal carcinoma. Data of this study suggest that Cripto-1 overexpression is connected with the tumorigenesis and progression of esophageal carcinoma; shRNA might be feasible for the inhibition of the invasion and metastasis of esophageal carcinoma.

## 1. Introduction

Esophageal cancer is the eighth most common cancer globally and the sixth most common cause of cancer mortality [1–3]. Esophageal cancer usually occurs as either esophageal squamous cell carcinoma (ESCC) in the upper two-thirds of the esophagus or as an esophageal adenocarcinoma (EAC) in the lower one-third of the esophagus. In the high-risk “esophageal cancer belt” (from northern Iran through central Asia to north-central China), 90% of esophageal cancer cases are ESSCs [1–3]. Likely, on account of increased tobacco and alcohol use, the incidence of ESCC has been increasing in this region, making ESCC the most dominant variety of esophageal cancer in Asia, especially in China (including Hong Kong and Taiwan), Korea, and Japan [1–3].

As ESCC is a highly aggressive carcinoma with a poor long-term outcome, surgical resection is considered the best treatment option; however, long-term survival after resection is approximately 25% in most Western studies, and the five-year survival after resection in Chinese studies is around 35% [4]. Thus, a better understanding of the pathophysiological mechanisms underlying ESCC invasiveness and metastasis is required to improve patient outcomes.

To this end, the epithelial-mesenchymal transition (EMT) has been identified as one of the key mechanisms underlying ESCC tumor invasiveness and metastasis and has been clinically associated with poor prognosis [5]. EMT is characterized by a loss of epithelial characteristics and an acquisition of a mesenchymal state that reduces cell adhesion and better enables ESCC tumor cells to dissociate from the epithelial tissue and migrate [6]. Therefore, the study of the mechanisms underlying EMT should improve our understanding of ESCC invasiveness and metastasis and may aid in clinical prevention and targeted therapy research efforts.

Previous molecular studies on EMT have identified changes in cell morphology accompanied by changes in several transcription factors (e.g., Snail, Slug, Twist, CBF-A, and KAP-1), the calcium-dependent cell adhesion molecules epithelial cadherin (E-cadherin, E-cad) and neural cadherin (N-cadherin, N-cad), and the intermediate filament protein Vimentin (Vim) [6]. E-cad downregulation, N-cad upregulation, and Vim upregulation have been well-established in EMT and are considered key state markers of EMT [5].

Our previous experiments on EMT in the human ESCC cell line Eca-109 have also shown the significant upregulation of cripto-1 (CR-1, CRGF, teratocarcinoma-derived growth factor 1, TDGF1), an epidermal growth factor-CR-1/FRL-1/cryptic (EGF/CFC) gene family member that appears to be associated with reprogramming differentiated tumor cells into cancer stem cells through inducing EMT [7, 8]. CR-1 is upregulated in several malignant tumors, including gastric cancer, breast cancer, and ovarian cancer, while being downregulated in normal tissue [9–12]. CR-1 also shows promise as a tumor-specific biomarker for the early detection of carcinomas as well as a target for chemotherapeutic development [13, 14].

In this study, we aimed to elucidate CR-1's role in ESCC development. We initially identified significant CR-1 upregulation in 41 surgically excised ESCC tumors—this CR-1 upregulation was statistically correlated with the EMT state markers of E-cad downregulation, N-cad upregulation, and Vim upregulation. We also demonstrated that CR-1 protein expression was positively correlated with nodal metastasis, distal metastasis, and clinical stage. Based on this clinical evidence, we successfully transfected the human ESCC cell line Ea-109 with a pSilencer2.1/CR-1-shRNA-413 expression vector that effectively silenced CR-1 expression* in vitro*. We showed that this CR-1 interference treatment significantly reduced the proliferation, S-phase fraction (SPF), and migratory ability of ESCC cells, while significantly increasing their apoptotic activity. We also showed that this CR-1 interference treatment combined with paclitaxel/cisplatin combination chemotherapy significantly potentiated these anticancer effects. These results indicate that CR-1 could be a useful target for ESCC prevention and therapy.

## 2. Materials and Methods

### 2.1. Ethics Statement

The research was approved by the Institutional Review Board (IRB) of the First Affiliated Hospital of Chongqing Medical University (Chongqing, China). Informed written consent was received from all participants prior to surgery.

### 2.2. Specimen Collection

From January 2011 to December 2011, esophageal specimens (including ESCC tumor tissue, paraneoplastic tissue, and normal margin tissue) were obtained from 41 patients (26 males and 17 females, mean age: 60.5 years, age range: 40–80 years) that either underwent esophageal surgical resection (for stages I–III ESCC patients) or esophagogastroduodenoscopy-based biopsy (for stage IV ESCC patients) in the Department of Thoracic Surgery at the First Affiliated Hospital of Chongqing Medical University. No patient received chemotherapy or radiation therapy prior to surgery. All tumor and paraneoplastic specimens were pathologically diagnosed as cases of primary ESCC with normal margins. Resected noncancerous esophageal tissue samples (~5 cm from the tumor) were used as healthy control specimens. Within thirty minutes of sample collection, a sterile scalpel was used to cut the specimens into 100–200 mg segments, which were immediately washed in PBS, placed in vials over liquid nitrogen, and refrigerated at −80°C. All samples were fixed in 10% formalin; half of the samples were used for routine pathological diagnosis, and the other half were embedded in paraffin and sliced in 4 *μ*m thick slices.

### 2.3. Western Blotting of CR-1, E-Cad, N-Cad, and Vim in Tissue Samples

ESCC samples were homogenized in a standard lysis buffer and then sonicated to promote lysis. The samples were centrifuged at 12000 rpm at 4°C for 10 min. The supernatants were collected, and the protein amounts were quantified by the BAC method. Lysates containing equal amounts of protein were heated at 95°C in SDS sample buffer for 5 min and then electrophoresed on 10% denaturing sodium dodecylsulphate-polyacrylamide gel electrophoresis (SDS-PAGE), transferred to PVDF membranes (Millipore), blocked with 5% skimmed milk for 1 h at room temperature, and incubated overnight at 4°C with the following primary antibodies diluted according to the manufacturer's instructions: anti-CR-1 rabbit monoclonal antibody, anti-E-cad rabbit monoclonal antibody, anti-N-cad rabbit monoclonal antibody, and anti-Vim rabbit monoclonal antibody (Epitomics Co. Ltd.). After three 30 min washings in 100 mM Tris-HCl buffer (pH 7.5) with 150 mM NaCl and 0.05% Tween 20 (TTBS buffer), the membranes were incubated at 37°C for 60 min with HRP-conjugated secondary antibody (1 : 3000, Jackson). Finally, the proteins were detected with ECL and exposed to film (Kodak, USA). Quantification of the immunoreactive bands was obtained with Bio-Rad Quantity One software (Bio-Rad). Values obtained were normalized based on internal *β*-actin density values. The results are expressed as the mean ± SD from at least three experiments.

### 2.4. Immunohistochemistry of CR-1, E-Cad, N-Cad, and Vim in Tissue Samples

Tissue samples were fixed for 24 h in 4% paraformaldehyde at 4°C and then processed by paraffin embedding according to standard methods. Consecutive 4 *μ*m sections were performed using a rotary microtome (Leica RM 2135, Meyer Instruments, Houston, TX, USA) for hematoxylin and eosin (HE) staining, TUNEL, and histochemistry. Immunohistochemical staining was performed by using the SP immunohistochemistry kit according to the manufacturer's instructions. Briefly, sections were dewaxed with xylene and rehydration. Microwave antigen retrieval was performed at 95°C for 15 min by using 0.01 M citric acid buffer (pH 6.0). The sections were incubated with a 3% hydrogen peroxide solution for 10 min at room temperature. Following several washes in PBS (pH 7.4), sections were blocked with 10% goat serum and then incubated overnight with goat anti-rabbit secondary antibody diluted according to the manufacturer's instructions (Beyotime Co. Ltd.) at 4°C. After several washes with PBS, biotinylated anti-mouse IgG or anti-rabbit IgG (secondary antibody) was added at 37°C for 30 min. After several washes with PBS, horseradish peroxidase-labeled streptavidin was added followed by 30 min incubation time at 37°C. After additional washes with PBS, immunoreactivity was detected with a diaminobenzidine (DAB) staining kit, and sections were counterstained with hematoxylin and eosin (HE). The results are expressed as the mean ± SD from at least three experiments.

### 2.5. mRNA Expression of CR-1, E-Cad, N-Cad, and Vim by Reverse Transcription Polymerase Chain Reaction (RT-PCR) in Tissue Samples

After removal from −80°C, the tissue specimens were ground in a liquid nitrogen-precooled mortar into powder form in order to extract an appropriate amount of RNA (about 100 mg) by addition of RNAiso Plus pulp to transparency at room temperature, followed by centrifugation for 5 min at 12000 g at 4°C. The supernatant was transferred to a new Ep tube; chloroform was added (1 : 5 v/v) and shaked at room temperature for 5 min, followed by 12000 g at 4°C for 15 min. The Ep supernatant was transferred to a new tube; an equal volume of isopropanol was added upside down, thoroughly mixed at room temperature for 10 min, and then centrifuged at 12000 g at 4°C for 10 min. The supernatant was discarded, and the precipitate was washed with 1 mL of 75% ethanol for retention, naturally dried, and dissolved in a small amount of DEPC water sufficient to dissolve the precipitate and then stored at −80°C. Then, 2 *μ*L of extracted RNA was added to 68 *μ*L DEPC water, wind, and percussion mixed with protein and nucleic acid analyzer RNA mixture, and its OD value and concentration were recorded.

The RT-PCR reaction was conducted with the following 25 *μ*L PCR reaction system: 12.5 *μ*L premix column, 2 *μ*L template (cDNA), 1 *μ*L primer 1 (20 *μ*M), 1 *μ*L primer 2 (20 *μ*M), and 8.5 *μ*L sterile distilled water. The reaction solution underwent light oscillation mixing and centrifugation. Reaction conditions were 94°C for 3 min, 94°C for 30 s, 58°C for 30 s, and 72°C for 30 s. The primers for CR-1, N-cad, E-cad, Vim, and the reference standard GAPDH are listed in Supplementary Table 1 in Supplementary Material available online at http://dx.doi.org/10.1155/2015/421285.

After PCR amplification, 10 *μ*L PCR products were separated by electrophoresis in 2% (w/v) agarose gels at 80 V for 30 min, stained with ethidium bromide, and photographed under UV light. Photographs were scanned and the intensity of the bands was determined using Quantity One image analysis software (Bio-Rad). Values were normalized with GAPDH cDNA. The results are expressed as the mean ± SD from at least three experiments.

### 2.6. Construction and Screening of CR-1 shRNA Plasmid Expression Vectors

The DH5a strain of* E. coli* fed-batch grown on glycerol and the human ESCC cell line Eca-109 were purchased from the Cell Bank of Shanghai Institute of Cell Biology, Chinese Academy of Sciences. The siRNA target design tools were used to design three CR-1-shRNA recombinant plasmids (pSilencer2.1-CR-1-shRNA-21, pSilencer2.1-CR-1-shRNA-413, and pSilencer2.1-CR-1-shRNA-619) and an unrelated sequence shRNA plasmid standard as a negative control. The oligonucleotides were annealed and inserted into the BamHI and HindIII sites of the pSilencer2.1-U6 neo expression plasmid using T4 DNA ligase (Tianjin Purcell Biotech Co., Supplementary Figure 1). BamHI and HindIII digestion showed a single band, indicating that the recombinant plasmid can be cut with BamHI and HindIII (Supplementary Figure 2). The annealed products were frozen at −20°C or directly used for the subsequent experiments.

pSilencer2.1-CR-1-shRNA-21, pSilencer2.1-CR-1-shRNA-413, and pSilencer2.1-CR-1-shRNA-619 were transformed into competent* E. coli* DH5*α*. After selection on LB-amp agar plates,* E. coli* colonies containing the insert were cultured for 15 h in LB-amp liquid media at 37°C. The plasmids were isolated using a plasmid extraction purification kit (TianGen) and submitted for automated sequencing and comparison with published sequences (GeneBank Blast) (Supplementary Figure 3).

### 2.7. Western Blotting and FQ-PCR of CR-1, N-Cad, E-Cad, and Vim in CR-1-Silenced Cells

The Eca109 human ESCC cell line was cultured in RPMI-1640 medium (GIBCO, Carlsbad, CA, USA) containing 10% fetal bovine serum (FBS) and 1% antibiotics (i.e., 100 U/mL penicillin G, 100 *μ*g/mL streptomycin sulfate) in an atmosphere of 37°C in 5% CO_2_ and passaged by treating with 0.02% EDTA in phosphate-buffered saline (PBS) and 0.25% trypsin when achieving confluence.

Harvesting of Eca109 cells using trypsin was done 24 h prior to transfection and plated in six-well plates with RPMI-1640-10% FBS without antibiotics. Four micrograms of purified expression vectors containing either the CR-1 shRNA insert (pSilencer2.1-CR-1-shRNA-21, pSilencer2.1-CR-1-shRNA-413, and pSilencer2.1-CR-1-shRNA-619) or the negative-control mismatch sequence (Invitrogen, Carlsbad, CA, USA) was transfected into Eca109 cells. Fluorogenic quantitative polymerase chain reaction (FQ-PCR) was performed 48 h after transfection to assess the selectivity of CR-1 gene silencing effect. Based on FQ-PCR data, pSilencer2.1/CR-1-shRNA-413 was determined to be the most efficient plasmid at silencing CR-1 in Eca-109 cells (Supplementary Figure 4).

Then, three groups were constructed: a CR-1 interference group (transfected with pSilencer2.1/CR-1-shRNA-413 plasmid), a mock control group (transfected with an empty plasmid), and a negative control group (transfected with an unrelated sequence). Eca109 cells were transfected accordingly, and then Western blotting and FQ-PCR were performed to detect the expression of CR-1, N-cad, E-cad, and Vim. The results are expressed as the mean ± SD from at least three experiments.

### 2.8. Cell Proliferation Assay of Targeted CR-1 Silencing with and without Paclitaxel/Cisplatin Combination Therapy

Five groups were then constructed: a mock control group (transfected with an empty plasmid), a negative control group (transfected with an unrelated sequence), a CR-1 interference group (transfected with pSilencer2.1/CR-1-shRNA-413 plasmid), a drug group (treated with paclitaxel + cisplatin), and a CR-1 interference + drug group (transfected with pSilencer2.1/CR-1-shRNA-413 plasmid and treated with paclitaxel + cisplatin).

A clonogenic cell proliferation assay was applied to assay cell proliferation in the five groups. The logarithmic phase of each group was digested into single cells by pipetting, and the cells were suspended in RPMI-1640 medium with 10% FBS. The density of each cell suspension was set to a standard of 1000 cells/well by inoculation with 10 mL prewarmed culture medium (37°C) in six-well plate and gently rotated to evenly disperse the cells. The cultures were then incubated for two weeks (37°C, 5% CO_2_) until the appearance of visible colonies. The supernatant was discarded and rinsed twice with PBS. Then, 5 mL 4% paraformaldehyde was used to fix the cells for 15 min. Giemsa staining was then conducted for 20 minutes, followed by washing away the stain slowly with water and air-drying. The six-well plate was placed upside down, and a transparent grid overlay was used to count the clones by light microscopy (low magnification). The cloning efficiency was calculated by the following formula: colony formation rate = (number of clones/seeded cells) × 100%. The results are expressed as the mean ± SD from at least three experiments.

### 2.9. Flow Cytometry of Targeted CR-1 Silencing with and without Paclitaxel/Cisplatin Combination Therapy

Flow cytometry was applied to assess cell cycle phase proportion in the five groups. Briefly, the cells were washed with PBS 48 h after transfection. Trypsin-EDTA digestion was performed to form a single-cell dispersion to prevent cell clustering, and the cells were then dissolved in 1 mL complete medium and transferred to a 1.5 mL Ep tube followed by 2000 rpm centrifugation for 5 min, and the supernatant was discarded. Then, 1 mL PBS/tube was used to rinse the cells, followed by 2000 rpm centrifugation for 5 min, and the supernatant was discarded. 1 mL PBS/tube was used to resuspend the cells. 2 mL of 70% precooled ethanol was added to fix the cells overnight at −20°C. The 3 mL in each tube was divided into three 1 mL aliquots that were pipetted into three 1.5 mL centrifuge tubes followed by 2000 rpm centrifugation for 5 min, and the supernatant was discarded. Approximately 1 mL PBS was added to the remaining 0.5 mL liquid to resuspend the remaining cells. Cells were centrifuged at 2000 rpm for 5 min, and the supernatant was discarded. 1 mL PBS was added to each tube followed by 2000 rpm centrifugation for 5 min, and the supernatant was discarded. 0.5 mL propidium iodide (PI) dye (5 *μ*g/mL including RNase) was added to each sample in the dark at room temperature for 30 min. Flow cytometry was conducted in triplicate with the three tubes to assess cell cycle phase proportion.

The extent of apoptosis in the five groups was also evaluated by flow cytometry. Forty-eight hours after transfection, the culture medium was aspirated off. The cells were immersed once in PBS, and the PBS was then aspirated off. Cells were conventionally digested, followed by 800 rpm centrifugation for 5 min, and the supernatant was discarded. Cells were resuspended in PBS. A 100 *μ*L sample of this cell suspension was added to a new 1.5 mL centrifuge tube, and then 10 *μ*L Annexin-VR-PE was flick mixed into the cell suspension and placed on ice for 20 min in the dark. Then, 380 *μ*L 1x binding buffer was added and flick mixed, followed by addition of 10 *μ*L 7-AAD and flick mixing. The rate of apoptosis was detected by flow cytometry, specifically, fluorescence-activating cell sorting (FACS) with excitation wavelengths of 488 nm and 578 nm with a wavelength channel filter for the detection of PE fluorescence. Other wavelengths of greater than 670 nm were filter channel detected by 7-AAD. This experiment was repeated in triplicate.

### 2.10. Transwell Assay of Targeted CR-1 Silencing with and without Paclitaxel/Cisplatin Combination Therapy

Cell invasiveness in the five groups was examined by a Transwell culture system. Briefly, a Transwell membrane coated with Matrigel was used for invasion assay. Cells were conventionally digested, followed by 800 rpm centrifugation for 5 min, and the supernatant was discarded. Cells were resuspended in serum-free medium. Then, 100 *μ*L cell suspension was seeded onto the upper wells of precoated Transwells, and 600 *μ*L of RPMI-1640 medium with 10% FBS was added to the lower chamber. The Transwell was incubated (37°C, 5% CO_2_) for 48 h. After incubation, the cells on the upper well and the membranes coated with Matrigel were swabbed with a Q-tip, fixed with 4% formalin, and stained with Giemsa as earlier described. The cells that penetrated through the filter were counted in six randomly selected fields, and the mean number of cells per field was recorded. The results are expressed as the mean ± SD from at least three experiments.

## 3. Results

### 3.1. Correlates of CR-1, N-Cad, E-Cad, and Vim Expression

**Figure 1:**
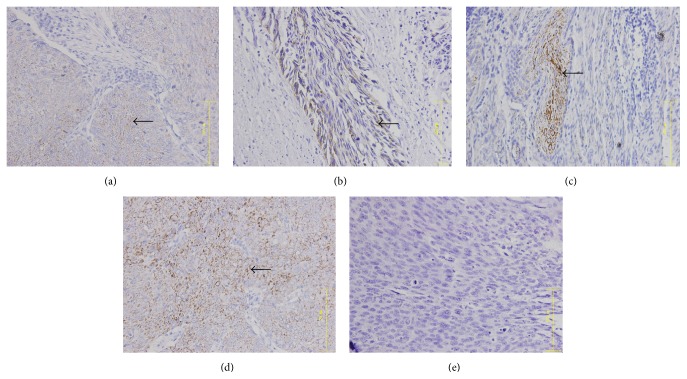
Cripto-1 expression in ESCC tumor samples. (a) Phase I, (b) phase II, (c) phase III, (d) phase IV, and (e) healthy control tissue. Arrow indicates CR-1+ cell. ×400 magnification.

**Figure 2 fig2:**
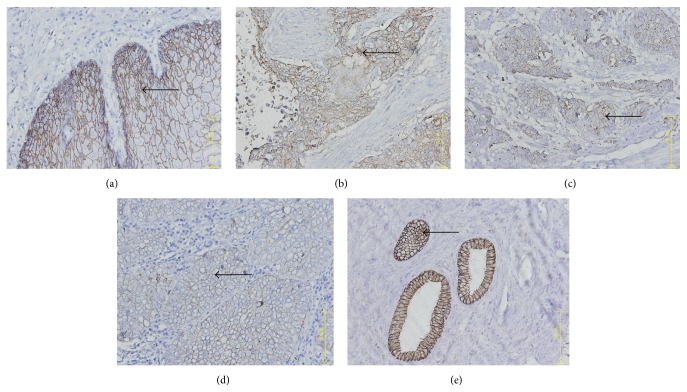
E-cadherin expression in ESCC tumor samples. (a) Phase I, (b) phase II, (c) phase III, (d) phase IV, and (e) healthy control tissue. Arrow indicates E-cad+ cell. ×400 magnification.

**Figure 3 fig3:**
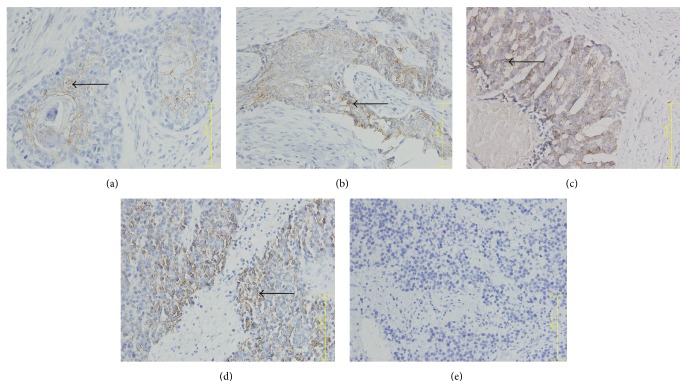
N-cadherin expression in ESCC tumor samples. (a) Phase I, (b) phase II, (c) phase III, (d) phase IV, and (e) healthy control tissue. Arrow indicates N-cad+ cell. ×400 magnification.

**Figure 4:**
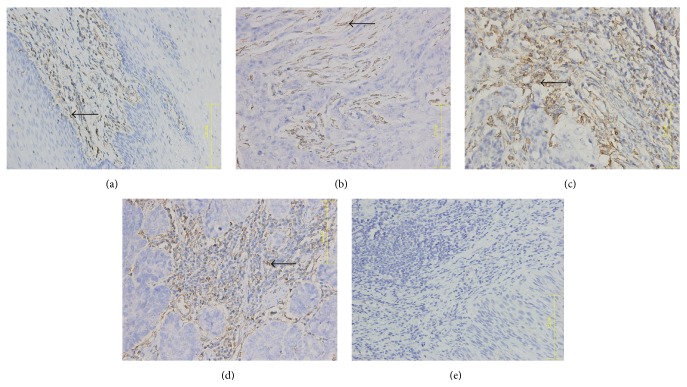
Vimentin expression in ESCC tumor samples. (a) Phase I, (b) phase II, (c) phase III, (d) phase IV, and (e) healthy control tissue. Arrow indicates Vim+ cell. ×400 magnification.

Immunohistochemistry showed that CR-1, E-cad, and N-cad expression mainly localized in the ESCC cell membrane and Vim expression mainly localized to the ESCC cytoplasm, while normal cells around the tumor margin were weakly positive or negative (Figures 1 [Fig fig2] [Fig fig3]–4).

The integrated OD values of CR-1, N-cad, E-cad, and Vim were used to calculate their average expression via IPP image processing software. CR-1, N-cad, and Vim expression in ESCC cells were positively correlated with disease occurrence and clinical stage (*r* = 0.989, *P* < 0.05), while E-cad expression in ESCC cells was negatively correlated with disease occurrence and clinical stage (*r* = −0.905, *P* < 0.05) (Tables 1 and 2).

CR-1 protein expression showed no significant correlation with gender or age (*P* > 0.05) (Table 3). CR-1 protein expression was positively correlated with nodal metastasis (*P* = 0.017), distal metastasis (*P* = 0.035), and clinical stage (*P* = 0.004).

### 3.2. Western Blotting and RT-PCR of CR-1, E-Cad, N-Cad, and Vim

**Figure 5:**
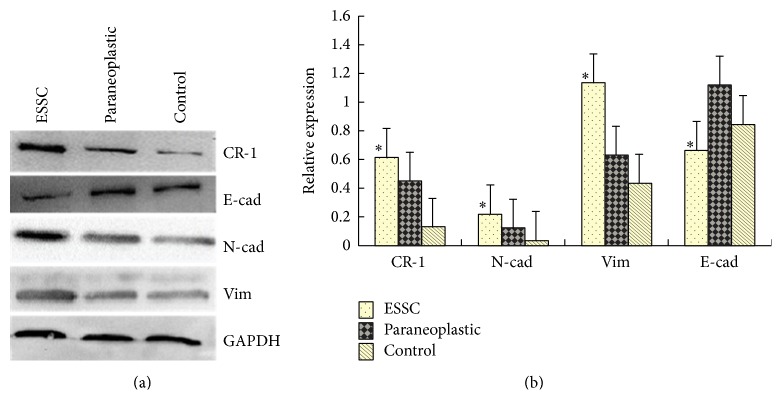
Protein expression of Cripto-1, E-cadherin, N-cadherin, and Vimentin in ESCC tumor samples by Western Blotting. (a) Western blots and (b) relative protein expression in ESCC, paraneoplastic, and healthy control tissues.

**Figure 6:**
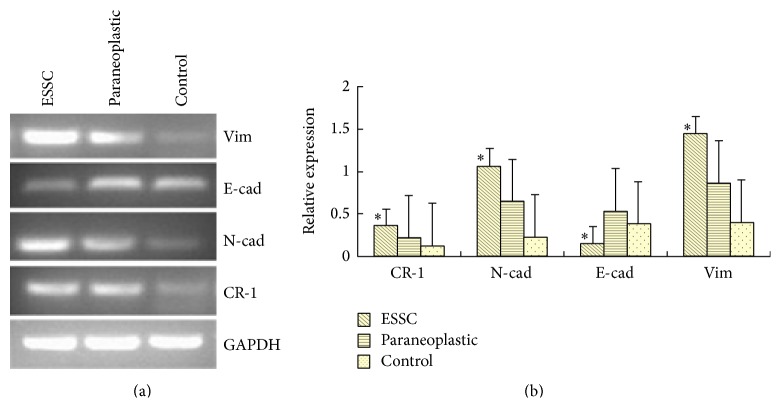
mRNA Expression of Cripto-1, E-cadherin, N-cadherin, and Vimentin in ESCC tumor samples by RT-PCR. (a) RT-PCR findings and (b) relative mRNA expression in ESCC, paraneoplastic, and healthy control tissues.

Western blotting revealed that ESCC cells showed significantly higher CR-1, N-cad, and Vim expression compared to paraneoplastic cells, which, in turn, showed significantly higher CR-1, N-cad, and Vim expression compared to healthy control cells (*P* < 0.05, Figure 5). In contrast, E-cad expression was significantly lower in ESCC cells as compared to healthy control cells, which, in turn, was significantly lower than paraneoplastic cells. RT-PCR revealed that the mRNA expression profiles of all four proteins were completely consistent with the Western blot findings (*P* < 0.05, Figure 6).

### 3.3. Postplasmid Transfection Western Blotting and FQ-PCR of E-Cad, N-Cad, and Vim

**Figure 7:**
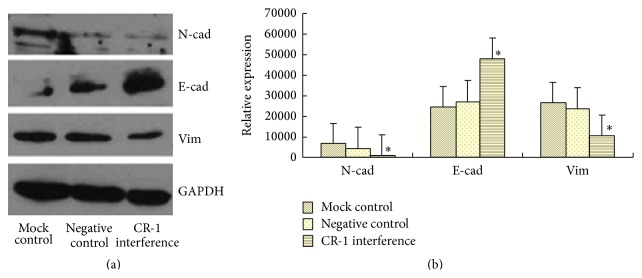
Posttransfection protein expression of E-cadherin, N-cadherin, and Vimentin by Western blotting. (a) Western blots and (b) relative protein expression in the mock control, negative control, and cripto-1 interference groups.

**Figure 8:**
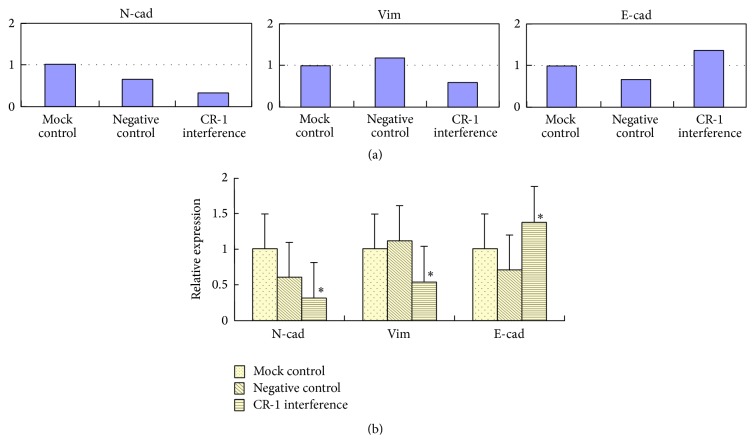
Posttransfection mRNA expression of E-cadherin, N-cadherin, and Vimentin by RT-PCR. (a) RT-PCR findings and (b) relative mRNA expression in the mock control, negative control, and cripto-1 interference groups.

After plasmid transfection of the Eca109 human ESCC cell line, Western blotting revealed that CR-1 interference significantly reduced N-cad and Vim protein expression, while significantly increasing E-cad expression, relative to the mock and negative control groups (*P* < 0.01, Figure 7). There was no significant differences between the two control groups (*P* > 0.05). FQ-PCR revealed that the mRNA expression profiles of all three proteins were completely consistent with the Western blot findings (*P* < 0.01), and there was no significant differences between the two control groups (*P* > 0.05, Figure 8).

### 3.4. Plasmid Transfection and Paclitaxel/Cisplatin Effects on Cell Proliferation

**Figure 9:**
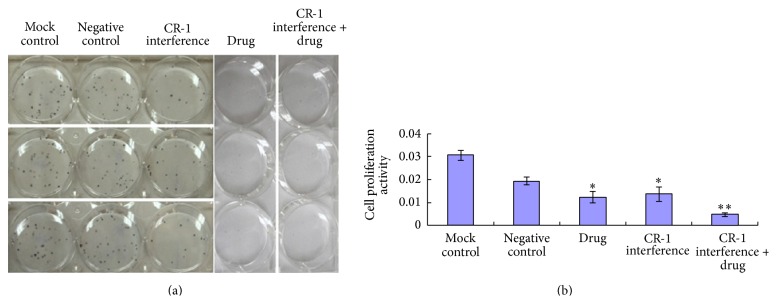
Cell proliferation assay. (a) Colony formation assay and (b) cell proliferation activity in the mock control, negative control, cripto-1 interference, drug, and cripto-1 interference + drug groups.

Cell proliferation in the CR-1 interference group, the drug group, and the CR-1 interference + drug group was significantly decreased relative to those of the mock and negative control groups (*P* < 0.01, Figure 9). Moreover, the CR-1 interference + drug group showed significantly decreased cell proliferation compared to both the CR-1 interference group and the drug group (*P* < 0.01). There was no significant differences in cell proliferation between the two control groups (*P* > 0.05).

### 3.5. Flow Cytometry of Cell Cycle Phase Proportion and Apoptotic Activity

**Figure 10:**
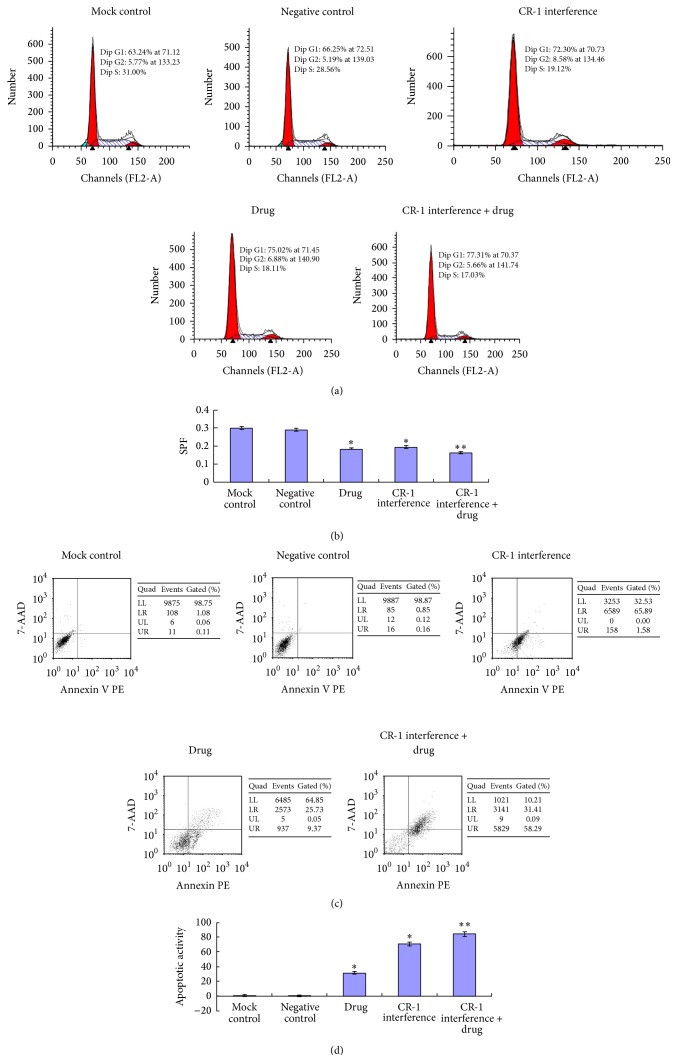
Flow cytometry. (a) Cell cycle phase detection by flow cytometry and (b) S-phase fractions of the mock control, negative control, cripto-1 interference, drug, and cripto-1 interference + drug groups. (c) Apoptosis detection by flow cytometry and (d) apoptotic activity of the mock control, negative control, cripto-1 interference, drug, and cripto-1 interference + drug groups.

The CR-1 interference group, the drug group, and the CR-1 interference + drug group displayed a significantly decreased S-phase fraction (SPF) relative to those of the mock and negative control groups (*P* < 0.01, Figures 10(a) and 10(b)). Moreover, the CR-1 interference + drug group showed a significantly decreased SPF compared to both the CR-1 interference group and the drug group (*P* < 0.01). There was no significant differences in SPF between the drug group and the two control groups (*P* > 0.05), and there was no significant differences in SPF between the two control groups (*P* > 0.05).

The apoptotic activity of the CR-1 interference group, the drug group, and the CR-1 interference + drug group was significantly increased relative to those of the mock and negative control groups (*P* < 0.01, Figures 10(c) and 10(d)). Moreover, the CR-1 interference + drug group showed significantly increased apoptotic activity compared to both the CR-1 interference group and the drug group (*P* < 0.01). There was no significant differences in apoptotic activity between the drug group and the CR-1 interference group (*P* > 0.05). There was no significant differences in apoptotic activity between the two control groups (*P* > 0.05).

### 3.6. Transwell Cell Migration Assay

**Figure 11:**
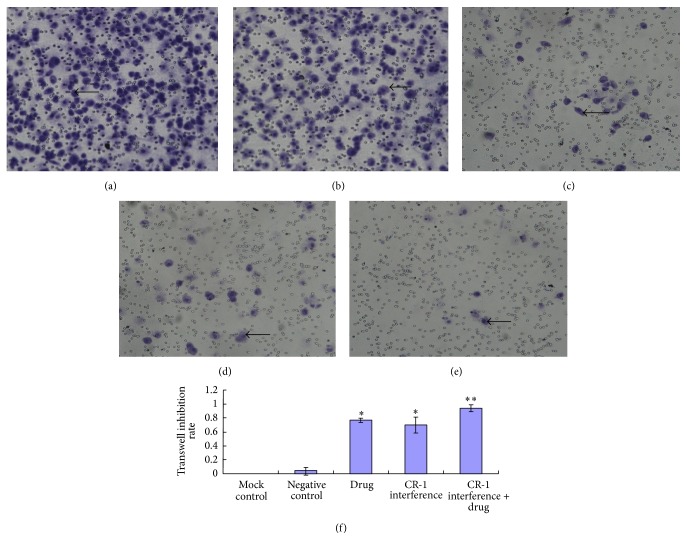
Transwell assay. (a) Mock control, (b) negative control, (c) cripto-1 interference, (d) drug, and (e) cripto-1 interference + drug groups. (f) Transwell inhibition rates of the mock control, negative control, cripto-1 interference, drug, and cripto-1 interference + drug groups.

The CR-1 interference group, the drug group, and the CR-1 interference + drug group displayed significantly increased inhibition of cell migration through the Transwell chamber relative to those of the mock and negative control groups (*P* < 0.01, Figure 11). The CR-1 interference + drug group showed a significantly increased inhibition of cell migration through the Transwell chamber compared to the CR-1 interference group and the drug group (*P* < 0.01). There was no significant differences in migratory activity between the drug group and the CR-1 interference group (*P* > 0.05). There was no significant differences in migratory activity between the two control groups (*P* > 0.05).

## 4. Discussion

ESCC is the result of multiple genetic and environmental factors that jointly participate in dysregulating a number of oncogenes and tumor suppressor genes [15]. Two traditional chemotherapeutics, paclitaxel (Taxol) and cisplatin (cis-diamminedichloroplatinum II), have been successfully used in ESCC patients [16]. Paclitaxel stabilizes microtubules and reduces their dynamicity (thereby promoting mitotic arrest and apoptosis) and has demonstrated strong results in the treatment of several early stage tumors, including esophageal cancer, lung cancer, cervical cancer, ovarian cancer, and prostate cancer [17]. Cisplatin forms platinum-DNA adducts that covalently cross-link between DNA strands, thereby inhibiting DNA replication and has been successfully used in various malignancies, including esophageal, gastric, ovarian, colorectal, and non-small-cell lung cancer [18].

As these two drugs act through different mechanisms, paclitaxel plus cisplatin combination chemotherapy is now commonly applied in the clinical treatment of ESCC [19]. Combination therapy is one of the basic principles of effective cancer chemotherapy, as it can be applied to obtain synergies, improve efficacy, reduce dosage, reduce toxic side effects, and delay drug resistance [20]. However, initial responders to combination therapy can eventually relapse due to acquired resistance, and tumor heterogeneity yields mixed response to combination therapy at different tumor sites [21]. Moreover, chemotherapy combinations reach a therapeutic plateau for metastatic disease [21]. To address these issues, investigators have focused on the combination of novel targeted agents together with traditional combination chemotherapy to optimize efficacy and survival and overcome acquired resistance [21].

Therefore, in this study, CR-1 interference treatment was combined with paclitaxel/cisplatin combination therapy to assess its anticancer effects on ESCC cells* in vitro*. We first identified significant CR-1 upregulation in surgically excised ESCC tumors from 41 ESCC patients and 41 healthy controls and demonstrated that CR-1 protein expression was positively correlated with nodal metastasis, distal metastasis, and clinical stage—this CR-1 upregulation was statistically correlated with the EMT state markers E-cad downregulation, N-cad upregulation, and Vim upregulation. Based on this clinical evidence, we then successfully transfected cultured human ESCC cells with a pSilencer2.1/CR-1-shRNA-413 expression vector that effectively silenced CR-1 expression. This CR-1 interference treatment was applied singularly (CR-1 interference group) and in combination with paclitaxel and cisplatin (CR-1 interference + drug group) to assess CR-1's effects on cell proliferation, cell cycle phase proportion, apoptosis, and migratory ability.

The mitogenic activity of CR-1 has been well-established. Overexpression of CR-1 or treatment with exogenous recombinant CR-1 induces cell proliferation in several cell lines [22]. In particular, the EGF-like domain of CR-1 has been shown to induce mitogenic signaling, and the mere addition of refolded peptides resembling the EGF-like domain of CR-1 has been shown to stimulate growth of several types of cancer cell lines [22]. CR-1 is believed to induce cell proliferation through multiple pathways, including ERK 1/2 activation, TGF-*β*/smad-2 signaling blockade, and TGF-*β*/activin B signaling blockade [22]. Accordingly, in this study, CR-1 interference treatment significantly reduced the proliferation of ESCC cells, illustrating that CR-1 gene expression promotes the proliferation of ESCC cells. Moreover, the CR-1 interference + paclitaxel/cisplatin combination therapy further reduced the proliferation of ESCC cells, suggesting a synergistic antiproliferative effect between CR-1 interference and these two drugs. Further studies should investigate CR-1's effect on ERK 1/2 activation, TGF-*β*/smad-2 signaling blockade, and TGF-*β*/activin B signaling blockade in ESCC cells.

Flow cytometry was then used to analyze cell cycle phase proportion and apoptotic activity in ESCC cells. CR-1 interference treatment significantly reduced the SPF while significantly increasing the apoptotic activity of ESCC cells. As the SPF is a measure of the percentage of cells that are in the phase of DNA synthesis (S-phase); CR-1 interference treatment significantly increases the proportion of cells arrested in the G2/M phase. These cell cycle and apoptotic findings are consistent, since G2/M phase arrest has been associated with induction of apoptotic signaling pathways [23]. Consistent with our findings, anti-EGF-like domain CR-1 antibodies produce apoptosis in cancer cells through significant reduction in activation of the AKT, c-Jun-NH2-terminal kinase, and p38 kinase signaling pathways [24]. Moreover, in this study, the CR-1 interference treatment + paclitaxel/cisplatin combination therapy further reduced the SPF while further increasing the apoptotic activity of ESCC cells, suggesting a synergistic effect between CR-1 interference and these two drugs in promoting G2/M phase arrest and apoptosis. In agreement with our findings, treating colon cancer cells with a combination of the rat anti-CR-1 antibodies and conventional cytotoxic drugs (e.g., 5-fluorouracil, epirubicin, or cis-platinum) results in a more significant inhibition of tumor cell growth compared with treatment with a single agent alone [24].

Previous studies have shown that the CR-1 overexpression plays an important role in the development of a more invasive mesenchymal phenotype [8]. Therefore, we applied the Transwell assay to assess the migratory ability of ESCC cells after CR-1 silencing. CR-1 interference treatment significantly reduced the migratory activity of ESCC cells, suggesting that CR-1 expression is associated with ESCC cell invasiveness. These findings, combined with our immunohistochemical results and CR-1's positive clinical correlations with nodal and distal metastasis and clinical stage, illustrate that CR-1 expression promotes ESCC cell invasiveness and metastasis. Moreover, the CR-1 interference treatment + paclitaxel/cisplatin combination therapy further reduced the migratory activity of ESCC cells, suggesting a synergistic effect between CR-1 interference and these two drugs in inhibiting ESCC cell invasiveness.

In conclusion, as CR-1 expression is positively associated with promoting the proliferation and invasiveness of ESCC cells, these findings signify that CR-1 acts as pathological factor in the EMT of ESCC cells. On this basis, CR-1 shows promise as a molecular drug development target for ESCC. However, further studies on the molecular mechanism(s) underlying CR-1's role in promoting the EMT in ESCC cells are warranted.

## Supplementary Material

Supplementary Figure 1. pSilencer2.1-U6 Neo Plasmid Map.
Supplementary Figure 2. Post-Digestion Electrophoresis of Recombinant Plasmids.
Supplementary Figure 3. Automated Sequencing of Recombinant Plasmids.
Supplementary Figure 4. Flurogenic Quantitative Polymerase Chain Reaction (FQ-PCR) of Cripto-1 mRNA Expression.
Supplementary Table 1. Reverse Transcription Polymerase Chain Reaction (RT-PCR) Primers.

## Figures and Tables

**Table 1:** Expression of Cripto-1, E-cadherin, N-cadherin, and Vimentin by immunohistochemistry.

Protein	ESSC	Control	*χ* ^2^
CR-1+	22 (53.7%)	8 (19.5%)	10.303^*∗*^
CR-1−	19 (46.3%)	33 (80.5%)	

E-cad+	12 (29.3%)	24 (58.5%)	7.13^*∗*^
E-cad−	29 (70.7%)	17 (41.5%)	

N-cad+	25 (61%)	11 (26.8%)	9.705^*∗*^
N-cad−	16 (39%)	30 (73.2%)	

Vim+	25 (61%)	13 (31.7%)	7.062^*∗*^
Vim−	16 (39%)	28 (68.3%)	

Chi-square test, ^*∗*^ *P* < 0.05.

**Table 2:** Mean integrated optical density of Cripto-1, E-cadherin, N-cadherin, and Vimentin.

Item	Control	Stage I	Stage II	Stage III	Stage IV
CR-1	1327.029	103306.90	105003.61	333017.38	425601.00
E-cad	8865.097	63116.79	218997.14	631183.81	502776.00
N-cad	5063.88	117223.75	171247.14	651362.06	476185.60
Vim	276815.2	307355.19	344192.69	119109.05	88986.17

**Table 3:** Cripto-1 protein expression and clinical data.

Clinical data	*N*	CR-1+ (%)	CR-1− (%)	*P* value
Gender				
Male	28	15 (53.5)	13 (46.5)	0.6260
Female	13	7 (53.8)	6 (46.2)	
Median age (yr)				
≤60	23	12 (52.2)	11 (47.8)	0.5400
>60	18	10 (55.6)	8 (44.4)	
T (infiltration range)				
T1-2	13	6 (46.2)	7 (54.8)	0.3740
T3-4	28	16 (57.1)	12 (42.9)	
N (nodal metastasis)				
N0	10	2 (20)	8 (80)	0.0200
N1-3	31	20 (64.5)	11 (35.5)	
M (distal metastasis)				
M0	36	17 (47.2)	19 (52.8)	0.0350
M1	5	5 (100)	0 (0)	
Clinical stage				
I-II	16	4 (25)	12 (75)	0.0040
III-IV	25	18 (72)	7 (28)	
