# Supplementary material for: Cripto-1 Promotes the Epithelial-Mesenchymal Transition in Esophageal Squamous Cell Carcinoma Cells
Source: Evid Based Complement Alternat Med. 2015 Sep 17;2015:421285. doi: 10.1155/2015/421285 (PMC4589627; doi:10.1155/2015/421285)
Supplement: Supplementary file 1 — Supplementary Figure 1. pSilencer2.1-U6 Neo Plasmid Map. Supplementary Figure 2. Post-Digestion Electrophoresis of Recombinant Plasmids. Supplementary Figure 3. Automated Sequencing of Recombinant Plasmids. Supplementary Figure 4. Flurogenic Quantitative Polymerase Chain Reaction (FQ-PCR) of Cripto-1 mRNA Expression. Supplementary Table 1. Reverse Transcription Polymerase Chain Reaction (RT-PCR) Primers. [file 421285.f1.zip › 421285.docx]

**Supplementary Figure 1. pSilencer2.1-U6 Neo Plasmid Map**

**Supplementary Figure 2. Post-Digestion Electrophoresis of Recombinant Plasmids**

BamHI and HindIII digestion of pSilencer2.1-CR-1-shRNA-413, pSilencer2.1-CR-1-shRNA-21, and pSilencer2.1-CR-1-shRNA-619 showed a single band, indicating that the recombinant plasmid can be cut with BamHI and HindIII.

**Supplementary Figure 3. Automated Sequencing of Recombinant Plasmids**

Sequences of (A) pSilencer2.1-CR-1-shRNA-413, (B) pSilencer2.1-CR-1-shRNA-21, and (C) pSilencer2.1-CR-1-shRNA-619.

**Supplementary Figure 4. Flurogenic Quantitative Polymerase Chain Reaction (FQ-PCR) of Cripto-1 mRNA Expression**

(A) FQ-PCR of cripto-1 mRNA expression. (B) pSilencer2.1/CR-1-shRNA-413 showed the highest efficiency in silencing cripto-1 mRNA expression in Eca-109 ESSC cells.

**Supplementary Table 1. Reverse Transcription Polymerase Chain Reaction (RT-PCR) Primers**
