# Supplementary figures and images for: Cripto-1 Promotes the Epithelial-Mesenchymal Transition in Esophageal Squamous Cell Carcinoma Cells
Source: Evid Based Complement Alternat Med. 2015 Sep 17;2015:421285. doi: 10.1155/2015/421285 (PMC4589627; doi:10.1155/2015/421285)

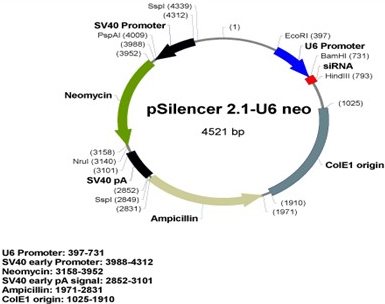

Supplement: Supplementary file 1 — Supplementary Figure 1. pSilencer2.1-U6 Neo Plasmid Map. Supplementary Figure 2. Post-Digestion Electrophoresis of Recombinant Plasmids. Supplementary Figure 3. Automated Sequencing of Recombinant Plasmids. Supplementary Figure 4. Flurogenic Quantitative Polymerase Chain Reaction (FQ-PCR) of Cripto-1 mRNA Expression. Supplementary Table 1. Reverse Transcription Polymerase Chain Reaction (RT-PCR) Primers. [file 421285.f1.zip › Supplementary Fig 1.tif]

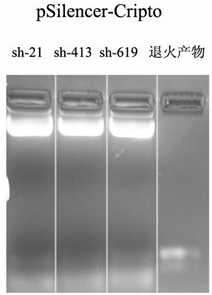

Supplement: Supplementary file 1 — Supplementary Figure 1. pSilencer2.1-U6 Neo Plasmid Map. Supplementary Figure 2. Post-Digestion Electrophoresis of Recombinant Plasmids. Supplementary Figure 3. Automated Sequencing of Recombinant Plasmids. Supplementary Figure 4. Flurogenic Quantitative Polymerase Chain Reaction (FQ-PCR) of Cripto-1 mRNA Expression. Supplementary Table 1. Reverse Transcription Polymerase Chain Reaction (RT-PCR) Primers. [file 421285.f1.zip › Supplementary Fig 2.tif]

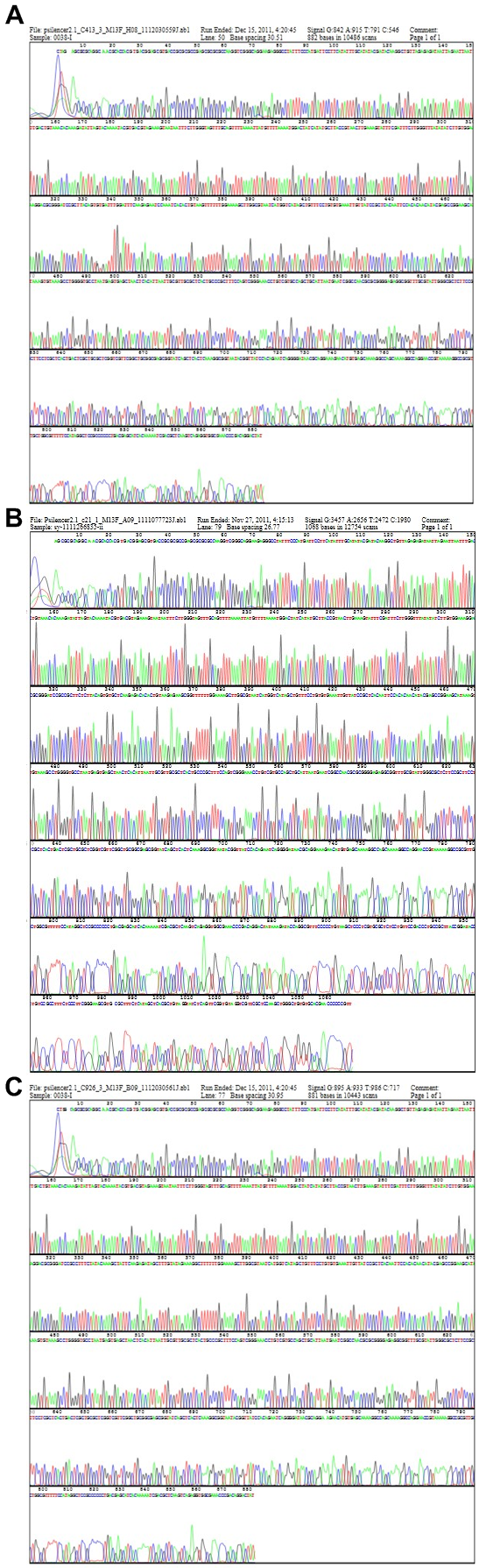

Supplement: Supplementary file 1 — Supplementary Figure 1. pSilencer2.1-U6 Neo Plasmid Map. Supplementary Figure 2. Post-Digestion Electrophoresis of Recombinant Plasmids. Supplementary Figure 3. Automated Sequencing of Recombinant Plasmids. Supplementary Figure 4. Flurogenic Quantitative Polymerase Chain Reaction (FQ-PCR) of Cripto-1 mRNA Expression. Supplementary Table 1. Reverse Transcription Polymerase Chain Reaction (RT-PCR) Primers. [file 421285.f1.zip › Supplementary Fig 3.tif]

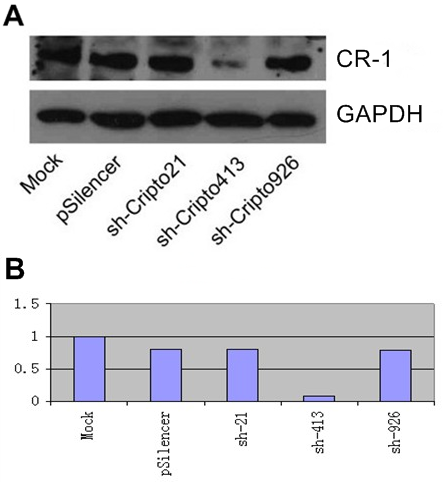

Supplement: Supplementary file 1 — Supplementary Figure 1. pSilencer2.1-U6 Neo Plasmid Map. Supplementary Figure 2. Post-Digestion Electrophoresis of Recombinant Plasmids. Supplementary Figure 3. Automated Sequencing of Recombinant Plasmids. Supplementary Figure 4. Flurogenic Quantitative Polymerase Chain Reaction (FQ-PCR) of Cripto-1 mRNA Expression. Supplementary Table 1. Reverse Transcription Polymerase Chain Reaction (RT-PCR) Primers. [file 421285.f1.zip › Supplementary Fig 4.tif]
