# Supplementary material for: Cripto-1 Promotes the Epithelial-Mesenchymal Transition in Esophageal Squamous Cell Carcinoma Cells
Source: Evid Based Complement Alternat Med. 2015 Sep 17;2015:421285. doi: 10.1155/2015/421285 (PMC4589627; doi:10.1155/2015/421285)
Supplement: Supplementary file 1 — Supplementary Figure 1. pSilencer2.1-U6 Neo Plasmid Map. Supplementary Figure 2. Post-Digestion Electrophoresis of Recombinant Plasmids. Supplementary Figure 3. Automated Sequencing of Recombinant Plasmids. Supplementary Figure 4. Flurogenic Quantitative Polymerase Chain Reaction (FQ-PCR) of Cripto-1 mRNA Expression. Supplementary Table 1. Reverse Transcription Polymerase Chain Reaction (RT-PCR) Primers. [file 421285.f1.zip › Tables.doc]

**Supplementary Table 1. Reverse Transcription Polymerase Chain Reaction (RT-PCR)** Primers

| **Item** | **Upstream primer** | **Downstream primer** | **Length** |
| --- | --- | --- | --- |
| CR-1 | CACGATGTGCGCAAAGAGA | TGACCGTGCCAGCATTTACA | 89bp |
| N-cad | ACTCGGCTTAATGGTGAT | CACTGGCAAACCTTCACG | 146bp |
| E-cad | CTTCTCTCACGCTGTGTCATC | CTCCTGTGTTCCTGTTAATGGT | 284bp |
| Vim | AGGCAAAGCAGGAGTCCA | TATCAACCAGAGGGAGTG | 375bp |
| GAPDH | AAGGTGAAGGTCGGAGTCAAC | GGGGTCATTGATGGCAACAAT | 102bp |
